# 285 Determinants of Mortality Amongst Adult Burn Patients Admitted to a Regional Burn Center: Twenty-year Review

**DOI:** 10.1093/jbcr/irad045.260

**Published:** 2023-08-29

**Authors:** Pankti Shah, Dhaval Bhavsar, Niaman Nazir, Jessica Reynolds, Maria C Vazquez Machado, Emily D Work

**Affiliations:** College of Osteopathic Medicine - Kansas City University, Lenexa, Kansas; University of Kansas Medical Center, Kansas City, Kansas; University of Kansas Medical Center, Kansas City, Kansas; The University of Kansas health system, Kansas City, Kansas; Ponce Health Science University, Ponce, Puerto Rico; Kansas University Medical Center, Olathe, Kansas

## Abstract

**Introduction:**

Established determinants of morbidity and mortality in burn patients include age and total burn surface area (TBSA), followed by inhalation injuries. We investigated determinants of mortality in burn patients admitted to our unit over the last two decades. The goal of this study was to determine the trends associated with burn mortality.

**Methods:**

The burn registry was reviewed for the data of 5,401 burn patients admitted to our burn unit from 2002 to 2021. Patient demographics, payer source, burn characteristics, TBSA, injury circumstances, length of stay, ventilator and ICU days, comorbidities, complications, and surgery/procedures data were obtained for all patients. Chart reviews were completed for patients who died to investigate trends in mortality. The chi-square test, Wilcoxon two sample Test, Cochran-Armitage trend test, and Jonckheere-Terptra test will be used to evaluate the difference in risk factors of patients who survived and those who died. Two-sided p values less than 0.05 were considered statistically significant.

**Results:**

A total of 4093 adult burn patients were hospitalized from 2002 to 2021, of which 155 died. Data showed a significant mortality risk for patients of older age with increased TBSA, higher number of ventilator days, higher ICU Days, and a higher number of complications and smoke inhalation. Male patients had a significantly higher mortality rate. Burn injury by flash-flame represented a higher mortality risk. Accidental burn injury unrelated to employment was significantly more common in all patient populations. The relationship between the number of comorbidities and mortality risk was not clear. There wasn’t a significant difference in the mortality risk based on ethnicity (p=0.1218). Mortality rates did not change significantly over the 20 years (p=0.2027).

**Conclusions:**

Age, %TBSA, and presence of inhalation injury continue to be major determinants of mortality. Higher incidence of longer stay and ICU/ventilator days, complications, and surgical procedures are associated with burn patients who don’t survive but these may be related to previous parameters. Overall mortality trends have not changed.

**Applicability of Research to Practice:**

The results of this study can be used to increase accuracy in the triage of burn patients at higher risk of complications and death, to offer patients precise prognostics and better outcomes in terms of morbidity and mortality.

